# Propensity score matching analysis to evaluate efficacy of polyethylene oxide adhesive on preventing delayed bleeding after gastric endoscopic submucosal dissection

**DOI:** 10.1038/s41598-022-08499-0

**Published:** 2022-03-16

**Authors:** Yang Yu, Tong Hu, Xiaoyi Kuai, Xiaoyu Liu, Rui Li, Chunli Zhou

**Affiliations:** 1 Department of Gastroenterology, The Affiliated Suzhou Hospital of Nanjing Medical University, Suzhou Municipal Hospital, Gusu School, Nanjing Medical University, Suzhou, Jiangsu China; 2 Department of Gastroenterology, The Second Hospital Yulin, Yulin, Shanxi China; 3 Department of Gastroenterology, The First Affiliated Hospital of Soochow University, Suzhou, Jiangsu China

**Keywords:** Oesophagogastroscopy, Outcomes research

## Abstract

Regardless of technical advancements, delayed bleeding is still a common adverse event after gastric endoscopic submucosal dissection (ESD), often occurring in the early postoperative phase. This study aimed to evaluate the efficacy of a newly designed polyethylene oxide (PEO) adhesive for preventing delayed gastric bleeding. Patients who underwent gastric ESD between December 2017 and December 2020 at three Chinese institutions were retrospectively reviewed. Patients receiving PEO application on gastric post-ESD ulcers were included in the PEO group, and patients without this procedure were included in the control group. To minimize potential bias, propensity score matching was performed, and sex, age, lesion size, lesion morphology, ulceration, localization, procedure time, frequency of major intraoperative bleeding, resected specimen size, lesion histopathology, submucosal invasion and the taking of antithrombotic drugs were included as matching factors. The incidence of delayed bleeding and time to bleeding were compared between both groups. After propensity score matching, 270 patients (135 per group) were included in the analysis. The delayed bleeding rate in the PEO group was significantly lower than that in the control group (1.5%, 2/135 vs. 8.9%, 12/135, *P* = 0.006). The median time (range) to bleeding was 4.5 (4–5) days in the PEO group and 2 (1–15) days in the control group, with no significant difference (*P* = 0. 198). PEO demonstrated a significant effect in reducing the rate of delayed bleeding. Further study is warranted to confirm the efficacy of PEO for bleeding that occurs in the early phase after gastric ESD.

## Introduction

The efficacy of endoscopic submucosal dissection (ESD) for treating gastric lesions has been well recognized^1–4^. However, except for an elevated en bloc resection rate, compared with the traditional endoscopic mucosal resection (EMR) technique, ESD induces a larger and deeper iatrogenic ulcer, which can lead to more adverse events^5,6^. Owing to advancements in endoscopic experience and perioperative management, the overall complication rate has decreased in recent years. However, although several preventive measures have been taken^7–9^, delayed bleeding remains a difficult problem^6,10^; according to meta–analysis, the pooled rate of delayed bleeding is approximately 5.1%^10^. Generally, bleeding of ESD-induced ulcers can be successfully managed by endoscopic haemostasis or conservative therapy^11^. However, in some cases, to avoid life-threatening haemorrhagic shock, surgical or interventional treatment is required^11–13^.

Many factors are thought to be associated with delayed bleeding^10,14–18^, and some are controversial^10^, such as different locations of the stomach^10,19^. However, the high incidence rate may be attributed to the large resected area and the use of antithrombotic drugs^6,8,11,14^. Currently, because of extended ESD indications^3^, both of these characteristics are becoming increasingly common. Thus, preventing delayed bleeding remains imperative. Most bleeding events occur within 48 h after ESD^10,20,21^; therefore, protecting early postoperative ulcers is highly cost effective and merits our attention.

Haemostatic powder is a kind of tissue adhesive designed for endoscopic use^22–24^. The substance is bibulous, and it achieves haemostasis by forming a protective gelled matrix layer on the wound surface^25,26^. In previous studies, its role in treating gastrointestinal bleeding has been proven valid^22,27–29^, especially when bleeding sites were located in difficult anatomical positions that conventional instruments could not reach^25,28^. Many studies have emphasized the short-term efficacy of haemostatic powder^23,25,27,28,31^; in some cases with massive haemorrhage, haemostatic powder was efficacious as a bridge to surgery. Moreover, a newly published study, which enrolled patients with large lesions and antithrombotic users, demonstrated that EndoClot polysaccharide haemostatic powder (PHP) had a tendency to decrease delayed bleeding in the early postoperative phase in high-risk patients^30^.

Apart from research on PHP and its preliminary study^25,30^, the current clinical experience regarding haemostatic powder mainly focuses on the area of treating gastrointestinal bleeding^22–24,27,28,31^. To obtain further information, we conducted this multicentre retrospective study, with the intention of evaluating the efficacy of the newly designed polyethylene oxide (PEO) adhesive on the prevention of delayed bleeding.

## Method

### Study design

This was a multicentre, retrospective, cohort study undertaken at three Chinese institutions with accumulated experience in endoscopic resection, including The Affiliated Suzhou Hospital of Nanjing Medical University, The First Affiliated Hospital of Soochow University, and Yulin Second Hospital. The study was approved in accordance with the Declaration of Helsinki by the Ethics Committee of each institution. Written informed consent was given to all participants for the ESD procedure and the application of PEO adhesive. ESD was performed in each centre by physicians with over 100 ESD procedures per year in the past 5 years.

### Patients

We retrospectively collected data based on medical records, endoscopic photos and videos from all consecutive patients at the above hospitals. All patients received ESD for gastric epithelial lesions between December 2017 and December 2020. An enhanced CT scan was performed to exclude lymph node or other organ metastasis before ESD. The inclusion criteria for eligible participants were as follows: (1) an age > 18 years. (2) Early gastric cancer or gastric adenoma within the expanded indication for endoscopic treatment. The exclusion criteria were as follows: (1) coagulopathy; (2) pregnancy; (3) multiple lesions; (4) additional surgery performed 30 days after ESD; (5) ESD ulcer surface fully sutured with a metal clip or other equipment; and (6) allergic history to PEO or other synthetic polymers. Patients receiving PEO application on post-ESD ulcers were included in the PEO group, and patients without this procedure were included in the control group.

### Management of antithrombotic drugs

Antithrombotic agents were classified into two major categories: anticoagulant and antiplatelet drugs. We consulted prescribing physicians or cardiologists and neurologists regarding the risk of thromboembolism during discontinuation of antithrombotic agents. Antithrombotic therapy was not stopped if patients had a high thromboembolic risk after their assessment. Additionally, heparin-bridging therapy was provided for warfarin users if needed. For patients without high thromboembolic risk, antiplatelet medication was discontinued 7 days before ESD, while anticoagulants were stopped 5 days prior to the procedure. The resumption of antithrombotic medication occurred on Day 7 after ESD.

### Equipment

A single-channel endoscope (GIF-HQ290, GIF-Q260J; Olympus, Tokyo, Japan) was used during ESD, and a transparent cap was attached to it to facilitate the procedure. A dual knife (KD-650U, KD-650L, Olympus) was used for marking and dissection. A pair of haemostatic forceps (FD-411UR, Olympus) was used to manage the remaining vessels on the ulcer floor. Other equipment included an Endoclip (ROCCD-26-195, Weichuang, Nanjing, China), injection needle (NM-200L-0423, Olympus), high-frequency generator (VIO 200D, ERBE, Tübingen, Germany), 0.2% indigo carmine dye (MICRO-TECH, Nanjing, China), and submucosal injection (a mixed solution of 250 mL normal saline solution + 1 mL indigo carmine + 2 mL norepinephrine). A CO_2_ insufflator (UCR, Olympus) was used for insufflation. As the last step, 3 g PHP adhesive (EndoClot Plus Co., Ltd., Suzhou, Jiangsu, China) was applied for post-ESD ulcers.

### ESD procedures

All patients fasted for at least 8 h before the procedure. ESD was performed under general anaesthesia as follows: (1) marking around the lesion; (2) submucosal injection; (3) mucosal circumferential incision and submucosal dissection; and (4) coagulation of visible vessels on the artificial ulcer bed. The procedure time was calculated from the beginning of the marking to the completion of lesion removal. The duration of PEO application was not counted towards procedure time. The definition of major intraoperative bleeding was arteriolar bleeding or diffuse venous bleeding in which haemorrhagic sites could not be located for the first time.

### PEO application

**Figure 1:**
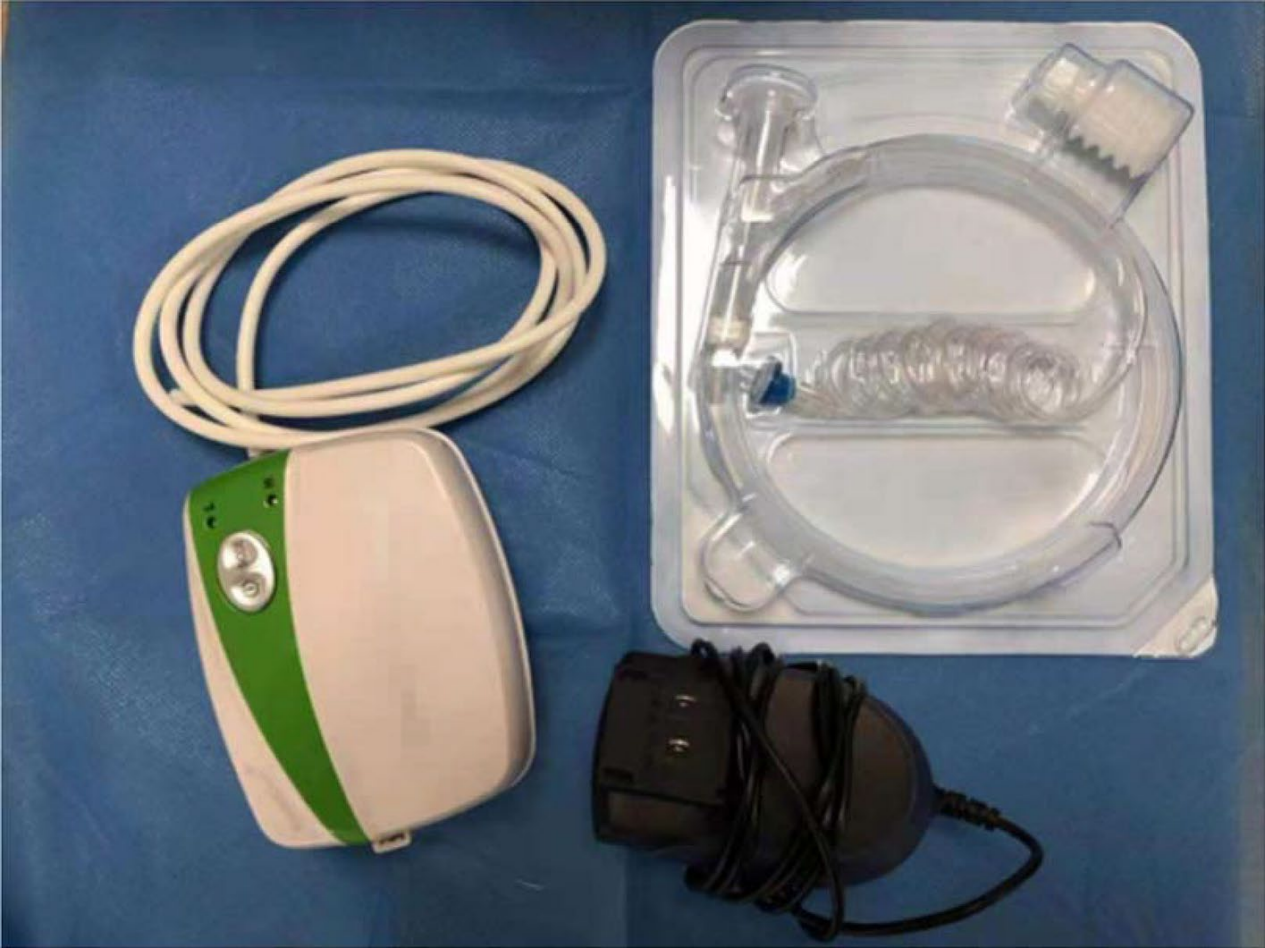
Photograph of the PEO application devices. ^1^PEO polyethylene oxide.

**Figure 2:**
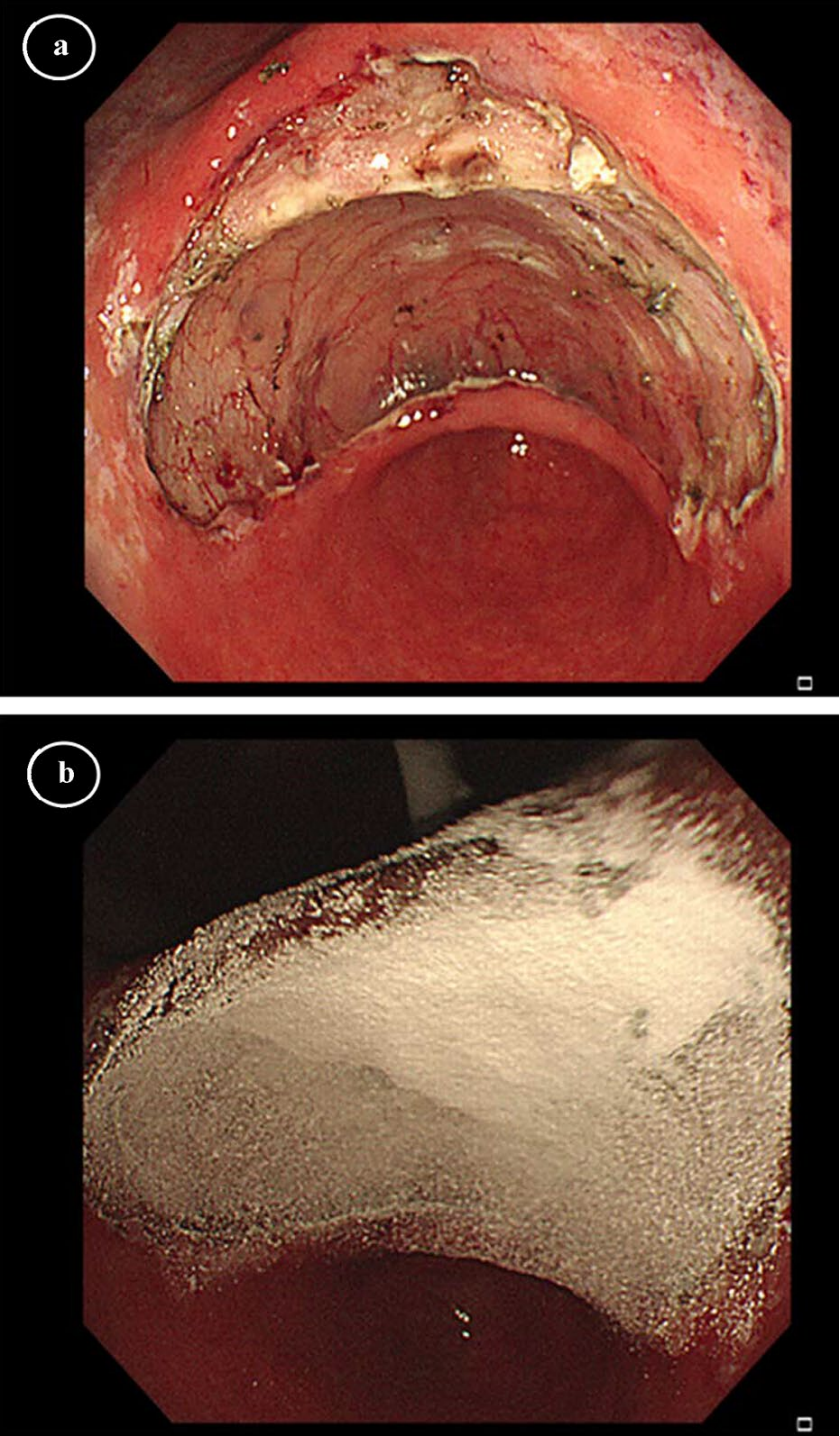
(**a**) A post-ESD ulcer located in the gastric angle with a longest diameter of 54 mm. (**b**) PEO adhesive applied to the ulcer surface. *ESD* endoscopic submucosal dissection, *PEO* polyethylene oxide.

A photograph of the EndoClot application devices is shown in Fig. 1. To ensure a standard procedure of PEO application, brief training (less than 30 min) was provided in each centre for both physicians and nurses before the first use. The decision to apply PEO adhesive was only made by surgeons during procedures. Usually, the powder was only sprayed for lesions that were more likely to develop delayed bleeding according to the physicians' judgement. After routine haemostasis and management of residual vessels, the PEO adhesive was applied to the ESD wounds via a catheter inserted into the endoscope's working channel (Fig. 2). A controllable airflow was generated by a portable air compressor to facilitate spraying. There were two modes for selection. A high flow of air was used during the insertion phase to dispel moisture. After the tube was targeted to the ulcer surface, the assistant altered the mode to a low flow of air. Simultaneously, the powder chamber was upended and knocked gently so that the PEO adhesive would evenly distribute on the wounds by the force of gravity and compressed air.

### Postoperative management and follow-up after ESD

All patients received a proton pump inhibitor (esomeprazole, 80 mg/day) via intravenous drip on the first 2 days after ESD. Subsequently, oral PPI administration (rabeprazole 20 mg/day) was provided for at least 2 months. The retention of gastric tubes was based on the surgeons' judgement. Usually, a fluid diet was started for all patients, and the tube was removed 48 h after the procedure. Considering the underlying bleeding risks, a scheduled second-look endoscopy was not performed in this study, and all patients were discharged if there was no sign of delayed bleeding or other complications on the fifth postoperative day (POD). Delayed bleeding was defined as haematemesis, melena or a remarkable haemoglobin decrease (> 2.0 g/dL) compared with the preoperative level, in which case urgent endoscopy was performed. Both groups were routinely followed up at outpatient clinics in the first week after ESD, and routine blood examination, liver and kidney function, and coagulation function were tested at that time. Postoperative laboratory indices were compared with the level of preoperative examination for each individual.

### Propensity score matching

**Figure 3:**
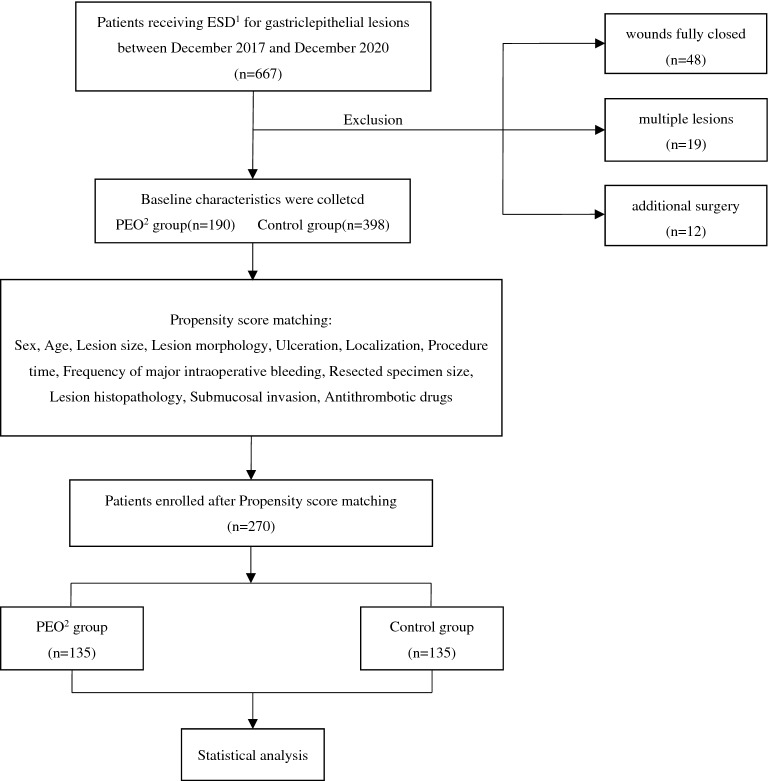
Flow diagram of patient selection for propensity score matching. ^1^*ESD* endoscopic submucosal dissection, ^2^*PEO* polyethylene oxide.

To compensate for potential confounding biases between the two groups, we performed propensity score matching. The flow diagram of patient selection for matching is shown in Fig. 3. The propensity-score model was estimated using a logistic regression model that adjusted for variables including sex, age, lesion size, lesion morphology (elevated vs. flat or depressed), localization (lesser curvature vs. others), procedure time, frequency of major bleeding, resected specimen size, lesion histopathology (adenocarcinoma vs. adenocarcinoma), ulceration, submucosal invasion and the taking of antithrombotic drugs. In addition, 1:1 matching with a calipre width of 0.02 was performed using nearest-neighbour matching without replacement. To determine the predictive power of propensity score model, the area under curve (AUC) of the receiver operating characteristic curve (ROC) was calculated, the matching model would be considered to be inappropriate if the AUC value was less than 0.8.To test the balance of covariates in matched pairs, a standardized difference less than 0.1 was set as an indication of being well balanced.

### Statistical analysis

After the normality test, skewed distributed variables were presented as medians and ranges. Comparisons between continuous and nonnormally distributed data were performed using the Mann–Whitney test. Meanwhile, the χ2 test or Fisher's exact test was conducted to compare differences between categorical variables. Factors associated with delayed bleeding were studied with univariate analysis. Odds ratios (ORs) and 95% confidence intervals (CIs) were calculated. P < 0.05 was considered to be statistically significant. All statistical analyses were performed with SPSS software, version 24.0 (SPSS Inc., Chicago, Ill).

## Results

### Patient and bleeding characteristics before propensity score matching

A total of 667 consecutive patients who received ESD for gastric epithelial lesions between December 2017 and December 2020 were initially enrolled. Among these subjects, 48 patients' wounds were fully closed with metal clips or other instruments, 19 patients with multiple lesions simultaneously treated by ESD and 12 patients who underwent additional surgery 30 days after ESD were excluded. Thus, 588 patients were included in the present study. The baseline characteristics of patients, lesions and procedural details between the PEO group (n = 190) and the control group (n = 398) are shown in Table 1. ESD was completed for all patients with an en bloc resection rate of 96.9% (570/588) and a curative resection rate of 96.1% (565/588). In comparison with the control group, there were more patients taking antithrombotic drugs (*P* = 0.049), and the PEO group had a significantly larger lesion (P < 0.001) and resected specimen (P < 0.001) size, together with a longer procedure time (P < 0.001). The times of major bleeding and preventive coagulation were more frequent in the PEO group (P < 0.001).

**Table 1:** Baseline characteristics of patients before propensity score matching.

Variables	PEO group	Control group	*P*	*SD*
**Patients**	n = 190	n = 398		
Age (range, years)*	65 (47–79)	65 (36–81)	0.113^1^	0.201
Gender (male/female)*	97/93	193/205	0.561^2^	0.051
Comorbidity				
Hypertension	94 (49.5%)	186 (46.7%)	0.534^2^	
Diabetes mellitus	78 (41.1%)	142 (35.7%)	0.208^2^	
Cardiopathy	36 (18.9%)	59 (14.8%)	0.204^2^	
Chronic kidney disease	19 (10.0%)	29 (7.3%)	0.261^2^	
Cerebrovascular disease	33 (17.4%)	61 (15.3%)	0.528^2^	
Patients taking antithrombotic drugs*	40 (21.1%)	58 (14.6%)	0.049^2^	0.159
Aspirin	28 (14.7%)	43 (10.8%)	0.171^2^	
Clopidogrel	12 (6.3%)	21 (5.3%)	0.609^2^	
Anticoagulants	11 (5.8%)	12 (3.0%)	0.105^2^	
Multiple use of antithrombotics	11 (5.8%)	20 (5.0%)	0.698^2^	
Continued use of antithrombotics	21 (11.1%)	30 (7.5%)	0.157^2^	
**Lesions**				
Vertical localization				
Upper third	56 (29.5%)	95 (23.9%)	0.146^2^	
Middle third	64 (33.7%)	161 (40.5%)	0.114^2^	
Lower third	70 (36.8%)	142 (35.7%)	0.783^2^	
Horizontal localization (lesser curvature/other)*	78 (41.1%)	149 (37.4%)	0.400^2^	0.073
Lesion size (range, mm)	20 (12–64)	18 (3–58)	< 0.001^1^	
Lesion morphology*			0.305^2^	0.092
Flat/depressed	122 (64.2%)	238 (59.8%)		
Elevated	68 (35.8%)	160 (40.2%)		
Ulceration*	38 (20.0%)	63 (15.8%)	0.210^2^	0.104
Histopathology*			0.348^2^	0.084
Adenoma	58 (30.5%)	137 (34.4%)		
Adenocarcinoma	132 (69.5%)	261 (65.6%)		
Submucosal invasion*	12 (6.3%)	15 (3.8%)	0.168^2^	0.104
**Procedure**				
Specimen size (range, mm)*	45 (28–95)	38 (25–78)	< 0.001^1^	0.793
Procedure time (range, mm)*	78 (50–120)	67 (39–127)	< 0.001^1^	0.742
En bloc resection	7 (3.7%)	11 (2.8%)	0.545^2^	
Curative resection	9 (4.7%)	15 (3.8%)	0.579^2^	
Frequency of major intraoperative bleeding (range, times)*	2 (1–4)	1.5 (0–4)	< 0.001^1^	0.964
Frequency of preventive coagulation (range, times)	16 (8–24)	10 (4–22)	< 0.001^1^	

*Variables included in propensity score matching as matching factors.

^1^Mann–Whitney U test.

^2^χ^2^ test.

The rate of delayed bleeding in the PEO group was relatively lower than that in the control group, but the difference was not significant (2.6%, 5/190 vs. 5.8%, 23/398, *P* = 0.094). No bleeding events occurred within 48 h after ESD in the PEO group, while in the control group, bleeding after 48 h was 60.9% (14/23). Regarding onset time, bleeding events occurred later in the PEO group than in the control group. The median (range) time was 2 (4–10) days in the control group and 5 (1–15) days in the PEO group, but the difference was not significant (*P* = 0.082).

For patients with delayed bleeding, endoscopic haemostasis was successfully performed without the help of surgery or interventional treatment. Electrocoagulation was a principal method and was performed for all bleeding sites. Metal clips were used as combined therapy for 5 lesions. Immediate haemostasis was achieved in all cases, and no rebleeding events occurred later. In the PEO group, no residual PEO layer was observed on the ulcer surface during endoscopic haemostasis. One patient in the control group experienced haemorrhagic shock on POD1 with transient tachycardia and hypotension, and he also received a blood transfusion because of a decrease in haemoglobin from 101 to 62 g/L. After endoscopic haemostasis and conventional medical treatment, the patient's condition stabilized on the same day, and he was discharged on POD6.

### Details of PEO application

PEO application was successfully performed for all patients. The median time (range) for covering the post-ESD ulcers was 93 (55–191) s. The catheter was clogged by PEO granules in one case, in which the post-ESD ulcer was located on the anterior side of the gastric angle. After altering the catheter to a spare one, the application was completed with no need to unblock the endoscopic working channel. In 15 cases, the vision was obscured by moist particles attached to the endoscope, and operators had to pull it out and clean the lens with wet gauze. No adverse events associated with PEO application were recorded during the follow-up period. In the PEO group, the postprocedural level of routine blood examination and liver, kidney and coagulation function showed no evident fluctuation, and we found no significant difference between the post- and pre-ESD indices.

### Patient and bleeding characteristics after propensity score matching

To minimize selection bias, 135 pairs were created using propensity score matching with the variables mentioned above (Table 2). After matching, there was no significant difference in baseline characteristics between the two groups.In addition to ulceration (0.235) and resected specimen size (0.141), the SD values of most matching factors were less than 0.1.The AUC value after propensity score matching was 0.803.

**Table 2:** Baseline characteristics of patients after propensity score matching.

Variables	PEO group	Control group	*P*	*SD*
**Patients**	n = 135	n = 135		
Age (range, years)*	65 (50–79)	66 (46–81)	0.140^1^	0.028
Gender*	70/65	66/69	0.626^2^	0.054
Comorbidity				
Hypertension	60 (44.4%)	60 (44.4%)	1.000^2^	
Diabetes mellitus	53 (39.3%)	41 (30.4%)	0.125^2^	
Cardiopathy	25 (18.5%)	29 (21.5%)	0.543^2^	
Chronic kidney disease	11 (8.1%)	6 (4.4%)	0.210^2^	
Cerebrovascular disease	24 (17.8%)	26 (19.3%)	0.754^2^	
Patients taking of antithrombotic drugs*	25 (18.5%)	22 (16.3%)	0.630^2^	0.099
Aspirin	17 (12.6%)	15 (11.1%)	0.706^2^	
Clopidogrel	10 (7.4%)	7 (5.2%)	0.452^2^	
Anticoagulants	5 (3.7%)	6 (4.4%)	0.758^2^	
Multiple use of antithrombotics	7 (5.2%)	8 (5.9%)	0.790^2^	
Continued use of antithrombotics	12 (8.9%)	12 (8.9%)	1.000^2^	
**Lesions**				
Vertical localization				
Upper third	37 (27.4%)	30 (22.2%)	0.324^1^	
Middle third	46 (34.1%)	39 (28.9%)	0.359^1^	
Lower third	52 (38.5%)	66 (48.9%)	0.086^1^	
Horizontal localization (lesser curvature/other)*	54 (40.0%)	54 (40.0%)	1.000^2^	0.095
Lesion size (range, mm)	20 (12–64)	22 (8–58)	0.128^1^	
Lesion morphology*			0.367^2^	< 0.001
Flat/depressed	86 (63.7%)	93 (68.9%)		
Elevated	49 (36.3%)	42 (31.1%)		
Ulceration*	26 (19.3%)	25 (18.5%)	0.876^2^	0.235
Histopathology*			0.892^2^	0.029
Adenoma	38 (27.4%)	37 (27.4%)		
Adenocarcinoma	97 (71.9%)	98 (72.6%)		
Submucosal invasion*	7 (5.2%)	9 (6.7%)	0.606^2^	< 0.001
**Procedure**				
Specimen size (range, mm)*	43 (28–95)	44 (26–78)	0.940^1^	0.141
Procedure time (range, minutes)*	76 (50–105)	75 (55–127)	0.776^1^	0.003
En bloc resection	3 (2.2%)	4 (3.0%)	1.000^3^	
Curative resection	4 (3.0%)	8 (5.9%)	0.238^3^	
Frequency of major intraoperative bleeding (range, times)*	2 (1–4)	2 (1–4)	0.889^1^	0.009
Frequency of preventive coagulation (range, times)	15 (8–20)	14 (8–22)	0.134^1^	

*Variables included in propensity score matching as matching factors.

^1^Mann–Whitney U test.

^2^χ^2^ test.

^3^Fisher exact test.

**Figure 4:**
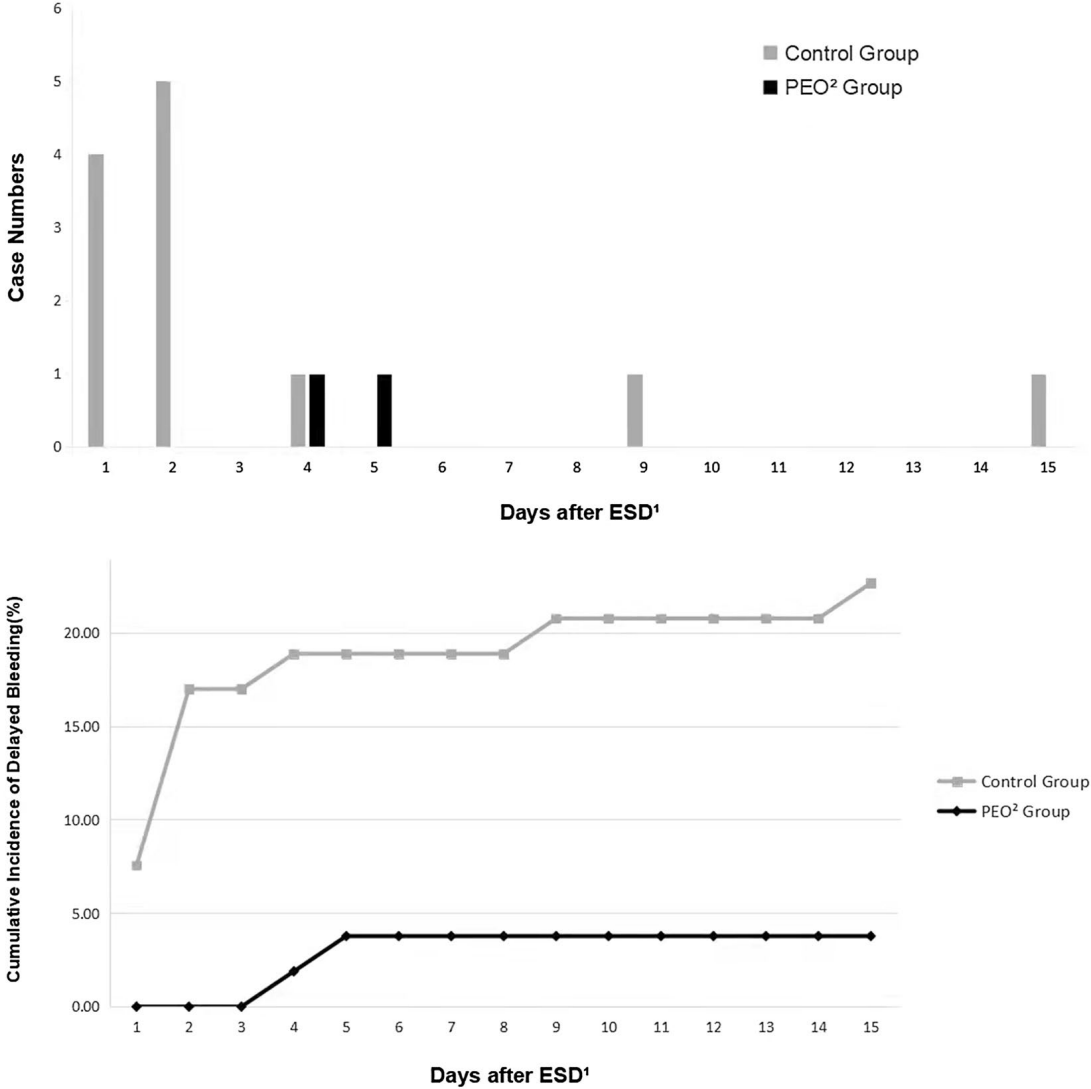
Case number and cumulative incidence of delayed bleeding after propensity score matching. ^1^*ESD* endoscopic submucosal dissection, ^2^*PEO* polyethylene oxide.

After propensity score matching, the rate of delayed bleeding was lower in the PEO group than in the control group, with a statistically significant difference (1.5%, 2/135 vs. 8.9%, 12/135, *P* = 0.006). In addition, bleeding events tended to occur later in the PEO group, and the median time (range) to bleeding was 2 (1–15) days in the PEO group and 4. 5 (4–5) days in the control group, and the difference was not significant (*P* = 0.198). The case number and cumulative incidence of delayed bleeding after propensity score matching are shown in Fig. 4. A majority of bleeding events in the control group occurred within 48 h, with a portion of 75.0% (9/12). The details of patients with delayed bleeding after propensity score matching are shown in the Supplementary Table.

### Factors related to delayed bleeding after propensity score matching

Univariate analysis (Table 3) showed that advanced age (≥ 65 years), the taking of antithrombotic drugs, continued use of antithrombotics and the frequency of major intraoperative bleeding were related to delayed bleeding. In contrast, the use of PEO was demonstrated to be a possible protective factor for delayed bleeding (OR: 0.154; 95% CI: 0.034–0.703; *P* = 0.016).

**Table 3:** Univariate analysis for delayed bleeding predictors after propensity score matching.

Variables	Univariate OR	95% CI	*P*
Age (≥ 65 years)	5.050	1.108–23.022	0.036
Male (female)	1.313	0.443–3.891	0.624
The taking of antithrombotic drugs	5.400	1.796–16.233	0.003
Multiple use of antithrombotics	3.115	0.631–15.393	0.163
Continued use of antithrombotics	6.930	2.110–22.754	0.001
Lesion size (increasing 1 mm)	1.036	0.977–1.099	0.235
Lesion location (vs. lesser curvature)	1.535	0.523–4.506	0.436
Lesion morphology (vs. flat/depressed)	0.777	0.237–2.549	0.677
Ulceration	0.704	0.153–3.248	0.653
Histopathology (vs. adenocarcinoma)	5.286	0.679–41.134	0.112
Submucosal invasion	1.236	0.151–10.090	0.843
Use of PEO	0.154	0.034–0.703	0.016
Specimen size (increasing 1 mm)	1.037	0.978–1.099	0.226
Procedure time (≥ 75: < 75)	1.070	0.361–3.174	0.902
Frequency of major intraoperative bleeding (increasing 1 time)	2.133	1.026–4.434	0.043

*OR* odds ratio, *CI* confidence interval.

## Discussion

This is the first multicentre study to evaluate the efficacy of PEO for delayed bleeding after gastric ESD. A total delayed bleeding rate of 4.8% (28/588) was reported, which is consistent with previous articles^10,11^. Owing to its retrospective nature, the present study was not as convincing as a randomized controlled trial (RCT). However, with the help of propensity score matching, we demonstrated that PEO was effective for protecting post-ESD ulcers in reducing the risk of delayed gastric bleeding.

PEO is a hydrophilic polymer in structure^32^ and is usually employed to extend drug release in pharmaceutical industries. The PEO adhesive and application system used in the present study were newly designed for endoscopic use. The powder is composed of small granules with a maximum water absorption ratio of 500. Once applied to the moist wound surface, an adherent layer is immediately formed. The gelled layer not only mechanically protects the wounds from acid erosion but also promotes topical aggregation of red blood cells, platelets and coagulation factors, accelerating the formation of blood clots.

In the present study, no clear indications, such as resection size and history of antithrombotics, were set for the application of PEO. However, the PEO group had a larger lesion and resected specimen size than the control group. Furthermore, the procedure time in the PEO group was longer, and more intraoperative bleeding occurred. Such variables have been recognized as risk factors for delayed bleeding in many studies^6,10,11,15^. Therefore, there were reasons to believe that the PEO group had a higher delayed bleeding risk.

The propensity score matching we performed was an attempt to compensate for differences and biases between the two groups. After matching, the PEO group showed a significantly lower rate of delayed bleeding than the control group. Thus, the efficacy of PEO adhesive in delayed bleeding prevention was demonstrated. By comparing the time to bleeding, we observed that bleeding events in the PEO group tended to occur later than those in the control group. Previous studies reported that haemostatic powder, for instance PHP, could only act on the ulcer surface for 3–48 hours^25,33,34^. Interestingly, no residue of the gelled layer was observed on post-ESD ulcers during endoscopic haemostasis, which was performed after POD 3. In contrast, early delayed bleeding occurred within 48 h, accounting for 75.0% (9/12) in the control group, which was consistent with the known features of delayed bleeding^10^. Obviously, PEO application altered the proportion of early and late delayed bleeding. Similar to our study, a recent Korean RCT^30^ found no bleeding up to POD7 in the PHP group. Thus, it could be speculated that PEO shields wounds during a short period and that the protective layer produced by PEO can remain on the ulcers for approximately 2 days. Therefore, PEO may be effective in preventing bleeding that occurs in the early phase after ESD, and with these effects, it further decreases the overall delayed bleeding rate.

A strong correlation was found between the use of antithrombotics and delayed bleeding by univariate analysis. Only two patients in the PEO group developed delayed bleeding after propensity score matching, and all of these patients continued to take antithrombotic agents during the perioperative period. According to previous studies^25,30^, the efficacy of haemostatic powder in patients who received antithrombotic treatment, especially with continued antithrombotic use, was unsatisfactory. This was a natural result because PEO is a kind of local haemostatic agent that cannot improve or influence coagulation function. The frequency of major intraoperative bleeding was another factor related to delayed bleeding, usually representing rich submucosal vessels and repeated electrocoagulation on the wound surfaces. As mentioned above, physicians were more inclined to apply PEO adhesive when lesions developed frequent intraoperative bleeding. Another well-recognized risk factor for delayed bleeding was resected specimen size^10,11,35^, which was not statistically significant according to the present study. There are two possible explanations. First, a resected specimen size larger than 40 mm was demonstrated by most studies as a risk factor for delayed bleeding^10,11,15,16,35^. However, the resection area may be larger in our study. After propensity score matching, the resected specimen size in both the PEO and control groups was larger than 40 mm, which made the statistics insignificant. Second, in contrast to antithrombotic drugs, resection size was more related to early-phase delayed bleeding^36–38^. However, as mentioned above, the application of PEO may decrease bleeding events that occur before POD 3. Therefore, to avoid excessive use of PEO, we suggest that it should be applied for patients with large post-ESD ulcers or repeated intraoperative bleeding. On the other hand, for patients without discontinuation of antithrombotic drugs, PEO application may be limited in the reduction of delayed bleeding rate.

As a bioadhesive polymer, PEO contains no allergens and can hardly be absorbed into blood. Underlying adverse events of haemostatic powder include intestinal obstruction and embolization, but none of these have been reported^22,25^. The relatively small usage amount owes a lot to this remarkable safety record. In this study, there were no PEO-related adverse events, and we found no significant difference in laboratory indices between the preoperative and postoperative levels. Thus, there were reasons to believe that PEO application was safe for shielding post-ESD ulcers.

As many have reported^22–25,28,30,31,39^, the noncontact powder application system is easy to use, and little experience or technical expertise is needed. From our experience, only 15–20 min of training is required before the first use of PEO. Application can be completed in 4 min, regardless of ulcer size and location in the stomach. Therefore, the use of PEO was convenient enough for physicians with little experience.

In the process of application, several disadvantages were found in the PEO and EndoClot systems. First, the vision was occasionally shaded and obscured by the scattering of PEO powder, which may have disturbed the spraying and caused an uneven distribution of the gelled layer. In one particular case, the clot formed by the powder and wet particles was too large to block the catheter. Fortunately, other types of medical adhesives may be able to overcome these limitations. Park et al.^40^ reported a new haemostatic adhesive powder (UI-EWD; Nextbiomedical, Incheon, South Korea) applied in patients with refractory upper gastrointestinal bleeding. UI-EWD achieved haemostasis by a unique coating technology, and no catheter clogging or scattering was reported during use.

Our study has some limitations. First, it was retrospective. Despite the propensity score matching we performed, our results were still not as convincing as would be the findings from RCTs because the selection bias could not be fully compensated for. Second, although undertaken at three medical centres, the sample size of this study was not large enough. In particular, the number of high-risk patients was insufficient. Thus, it was not only difficult to conduct further subgroup analysis with a limited sample size, but this also compromised the value of our results. Third, there have been some studies comparing the efficacy of different endoscopic haemostatic agents, with limited conditions, only one blank control group was set. Further prospective RCT with large sample sizes are required.

## Conclusions

In conclusion, the present study demonstrated a significant effect of PEO on the reduction of delayed bleeding after gastric ESD. Bleeding events tended to occur in later periods after PEO application, suggesting that PEO could effectively shield post-ESD ulcers and prevent bleeding occurring in the early phase after the procedure. Considering its convenience and safety, PEO may be a good candidate for post-ESD ulcer protection, although further RCTs on a large scale are warranted.

## Supplementary Information

Supplementary Table 1.
