# Supplementary material for: Propensity score matching analysis to evaluate efficacy of polyethylene oxide adhesive on preventing delayed bleeding after gastric endoscopic submucosal dissection
Source: Sci Rep. 2022 Mar 16;12:4538. doi: 10.1038/s41598-022-08499-0 (PMC8927376; doi:10.1038/s41598-022-08499-0)
Supplement: Supplementary file 1 — Supplementary Table 1. [file 41598_2022_8499_MOESM1_ESM.docx]

**Supplementary Table 1 The details of 14 patients who experienced delayed bleeding after propensity score matching**

| Case | Group | Age, | Specimen size, | Antithrombotic agents, | Major intraoperative bleeding, | Clinical manifestations | Onset, | Forrest classification | Drop in Hb level, | Haemostasis methods |
| --- | --- | --- | --- | --- | --- | --- | --- | --- | --- | --- |
| NO |  | years | mm | (continued use/with cessation) | times |  | POD |  | g/L |  |
| 419 | PEO | 67 | 42 | Warfarin (continued use) | 2 | haematemesis, melena | 4 | Ⅱa | 12 | Electrocoagulation |
| 452 | PEO | 68 | 38 | Aspirin (continued use) | 3 | melena | 5 | Ⅰb | 10 | Electrocoagulation, clipping |
| 10 | Control | 73 | 42 | Clopidogrel (continued use) | 2 | melena | 9 | Ⅱa | 22 | Electrocoagulation |
| 13 | Control | 72 | 46 | - | 2 | melena | 4 | Ⅱb | 15 | Electrocoagulation |
| 30 | Control | 67 | 36 | Aspirin (with cessation) | 1 | haematemesis | 1 | Ⅰa | 39 | Electrocoagulation, clipping |
| 84 | Control | 63 | 53 | - | 3 | melena | 2 | Ⅱa | 8 | Electrocoagulation |
| 131 | Control | 65 | 58 | - | 2 | melena | 1 | Ⅱa | 10 | Electrocoagulation |
| 206 | Control | 73 | 50 | - | 3 | melena | 15 | Ⅲ | 24 | Electrocoagulation |
| 213 | Control | 70 | 45 | - | 2 | haematemesis | 1 | Ⅰb | 6 | Electrocoagulation |
| 269 | Control | 65 | 55 | - | 3 | melena | 2 | Ⅱa | 13 | Electrocoagulation |
| 272 | Control | 71 | 48 | Aspirin (continued use) | 3 | haematemesis | 1 | Ⅰb | 11 | Electrocoagulation |
| 277 | Control | 73 | 47 | Clopidogrel (continued use) | 3 | melena | 2 | Ⅱa | 17 | Electrocoagulation |
| 279 | Control | 76 | 46 | Aspirin, warfarin (with cessation) | 2 | melena | 2 | Ⅲ | 8 | Electrocoagulation |
| 319 | Control | 53 | 40 | - | 3 | haematemesis, melena | 2 | Ⅰb | 15 | Electrocoagulation, clipping |

POD, postoperative day Hb, haemoglobin
